# Supplementary material for: Comparison of healthcare costs of patients with COPD on maintenance inhaled therapies between 2011 and 2019 in Hungary using a nationwide database
Source: PLoS One. 2025 May 5;20(5):e0320949. doi: 10.1371/journal.pone.0320949 (PMC12052116; doi:10.1371/journal.pone.0320949)
Supplement: S1 Appendix — (DOCX) [file pone.0320949.s001.docx]

**Comparison of healthcare costs of patients with COPD on maintenance inhaled therapies between 2011 and 2019 in Hungary using a nationwide database**

**Short title: Healthcare costs of COPD in Hungary**

Brigitta Dombai^1¶^, Viktória Nagy^2¶^, István Ruzsics^3^, László Németh^4^, Tamás Balázs^4^, Balázs Sánta^5&^, Zsófia Lázár^6&*^

^1^Outpatient Health Care Services of Kispest, Pulmonology Centre, Budapest, Hungary

^2^Department of Pulmonology, Géza Hetényi Hospital of Jász-Nagykun-Szolnok County, Szolnok, Hungary

^3^1^st^ Department of Internal Medicine, Division of Pulmonology University of Pécs, Medical School, , Pécs, Hungary

^4^Healthware Consulting Ltd., Budapest, Hungary

^5^Chiesi Hungary Ltd., Budapest, Hungary

^6^Department of Pulmonology, Semmelweis University, Budapest, Hungary

**Supporting information Health insurance and healthcare financing in Hungary**

The National Health Insurance Fund (NHIF) database is a complex repository that covers most of the Hungarian population, collecting healthcare-associated data, such as inpatient and outpatient visits, imaging and laboratory analyses, reimbursement for drug prescriptions, linking the data to the International Classification of Diseases (ICD-10) codes of these events. The data are connected to individual patients via their insurance numbers. Based on Act No. 80/1997 and Act No. 63/2012, the NHIF has the legal right to handle patients’ data, and to share it on a claim basis. Due to the protection of personal healthcare data, we cannot access data of individual patients or groups that include less than 10 people.

The NHIF plays a central role in the Hungarian healthcare system, as a sole public healthcare insurer. It has individual budgets for the following expenditures: inpatient care, outpatient care, medication, and medical aids. Outpatient costs cover visits at GPs or specialists, laboratory measurements and imaging. Medical aids prescribed for the ICD-10 code of J44 in Hungary are nebulizers, flutters, breathing trainers and medical home oxygen concentrators. Other prescription-restricted non-COPD medical aids include items such as continuous and biphasic positive airway pressure machines, materials for insulin administration, mobility devices, prostheses, medical hearing aids and equipment for home nursing.

Healthcare costs are fully or partially covered by NHIF. All costs of out- and inpatient care, are fully reimbursed, while certain percentage of costs of medication (e.g. for COPD inhaled maintenance medications: 90%, oxygen therapy: 100%) and medical aids (usually 50%) are covered. If medication is fully reimbursed by the NHIF, the patient still must pay 300 HUF (0.77 USD as of Nov 2024) for filling in a prescription.

Hungarian citizens with social security coverage become eligible for social security-covered healthcare services. Access to these services primarily depends on establishing a legal connection through work status, as insurance fees are generally automatically deducted from monthly salaries, so all people who are employed in Hungary are automatically eligible for insurance. Furthermore, all people under 16 years of age, and all full-time students (including high school, university or other educational institutions) below or above 18 years of age, all pensioners are insured. Finally, unemployed people are also eligible to apply for free insurance. Citizens without social security coverage are obliged to pay a healthcare service contribution fee. Medical services not covered by social security are available but represent a negligible share.

Hungary is the first European country that used the Diagnosis Related Group (DRG) financing system for reimbursing hospitals nationwide. A DRG system provides categories for grouping patients with similar characteristics, based on their diagnoses. For each group, resource use is estimated and translated to a fixed amount of reimbursement for the hospital.

**Patients**

Eligible patients were over 40 years old, received COPD maintenance inhaled therapy i.e. long-acting muscarinic antagonists (LAMA) or long-acting beta2-agonists (LABA) alone or in combination, or together with inhaled corticosteroids (ICS), prescribed with J41-44 ICD-10 codes, which are detailed in Table 1.

**Calculation of costs of long-term oxygen therapy**

The use of long-term oxygen therapy was defined by prescriptions for a home oxygen concentrator, liquid oxygen, or oxygen gas, prescribed for ICD-10 codes of COPD and/or chronic respiratory insufficiency, reimbursed as medication or medical aids. We presumed that these products were indicated due to chronic respiratory insufficiency related to COPD in all patients.

**Trends in the prices of the most common inhaled maintenance drugs for COPD**

Many basic therapeutical costs were price-controlled by NHIF in the study period and were not adjusted to the rate of inflation. Specifically, here we show in Table 2 that there was no increase in the total prices of the most frequently prescribed maintenance inhaled medications for COPD using publicly available information. Indeed, due to the appearance of generic drugs, the price of some drugs has declines (e.g. the LAMA tiotropium).

**Statistical analysis**

Descriptive statistics were calculated for each calendar year using the variables mentioned above. For modelling the annual costs, a generalized linear mixed regression^1^ was applied using Gamma distribution with logarithmic link function. Considered covariates were age, sex, calendar year, status of incidence, place of residence, CCI, number of hospitalization and number of days covered by maintenance inhaled therapy. The year 2011 was excluded from the modelling process to reduce bias of the incidence variable (as every patient was an incident patient in that year). Linear relationships between age and the number of hospitalizations or the days covered by maintenance inhaled therapy were considered too rigid for the model as a one-unit difference has generally less effect at larger values than at smaller values. As non-linear relationships were assumed for these covariates, quadratic terms were also used in the model, i.e. the linear and quadratic terms together determined the effect (multiplicative effect as shown in Figures 1-4). The linear term had a stronger effect on estimates around zero, while the quadratic term was responsible for the behaviour of higher values. In case of a negative quadratic terms, the functions had peaks. The glmmTMB^2^ and DHARMa^3^ R packages were used for model fitting and diagnostics.

After model fitting, also model diagnostics were performed for all-cause total, all-cause hospitalizations, COPD-related total and COPD-related hospitalizations costs (Figure 5). The figures are based on the DHARMa R package and illustrate the distribution of standardized residuals based on the predicted values. For optimal models, the quantile lines should be horizontal and equally spaced. This somewhat held for all-cause hospitalization costs and COPD-related hospitalization cost when the prediction was not extremely high (left side of figure). There were waves in the diagnostics of total costs, especially at higher regions. It may have been caused by the general conditions of the patients that can be measured by labour values, but unfortunately in financial database these were not available. All the diagnostic figures had numerous extreme values (upper and lower edges of the figure), which is common in cost analysis. In general, the diagnostics did not yield perfect residual plots, but the results of these models were well-established.

An additional sensitivity analysis was performed on a representative sample of 20,000, using random sampling. The Charlson comorbidity index was replaced by individual comorbidities (Table 3) and their variance inflation factor (VIF) values were calculated in different models (Table 4). In addition to the original model, a model containing individual comorbidities, as well as models without adjusting the number of maintenance inhaled therapy days, the number of hospitalizations, or both, were also examined. The models were compared based on their AIC values (Table 4). Model fit was best in the second model, where individual comorbidities were also considered. However, the variables related to the calendar year showed similar behavior here as in the original model. Excluding the number of maintenance inhaled therapy days or the number of hospitalizations resulted in worse fitting models, except for the case of COPD-specific inpatient costs, where the fit of the original and the model without adjusting to the number of maintenance inhaled therapy days was similar. Based on the model fit and the coefficient values, we decided to report the original model, as it was also available in full sample size. The coefficients of the models with individual comorbidities obtained in the subsample are shown in Figures 6-9.

**References**

1. Faraway JJ. Extending the Linear Model with R: Generalized Linear, Mixed Effects and Nonparametric Regression Models. Boca Raton: Taylor & Francis Group, LLC; 2006.

2. Brooks ME, Kristensen K, van Benthem KJ, Magnusson A, Berg CW, Nielsen A, et al. glmmTMB Balances Speed and Flexibility Among Packages for Zero-inflated Generalized Linear Mixed Modeling. The R Journal. 2017; **9**:378–400. https://doi.org/10.32614/RJ-2017-066

3. Hartig F. DHARMa: Residual Diagnostics for Hierarchical (Multi-Level/Mixed) Regression Models. R package version 0.4.4. 2022 https://CRAN.R-project.org/package=DHARMa

**Figure captions**

**Figure 1. The effects of hospitalization frequency, age and therapy-covered days on total all-cause healthcare costs**

**Figure 2. The effects of hospitalization frequency, age and therapy-covered days on the costs of all-cause hospitalizations**

**Figure 3. The effects of hospitalization frequency, age and therapy-covered days on total COPD-related healthcare costs**

**Figure 4. The effects of hospitalization frequency, age and therapy-covered days on the costs of COPD-related hospitalizations**

**Figure 5. Model diagnostics for all-cause total, all-cause hospitalizations, COPD-related total and COPD-related hospitalization costs.**

**Figure 6. The association of clinical variables and individual comorbidities to all-cause total healthcare costs.**

**Figure 7. The association of clinical variables and individual comorbidities to all-cause inpatient healthcare costs.**

**Figure 8. The association of clinical variables and individual comorbidities to COPD-specific total healthcare costs.**

**Figure 9. The association of clinical variables and individual comorbidities to COPD-specific inpatient healthcare costs.**

| **Table 1. Maintenance inhaled therapies of COPD used to assess the eligibility of patients and to determine the inhaled drug regimens** | | | |
| --- | --- | --- | --- |
| **Drug class** | **Active drugs** | **Brand names** | **Days of therapy (DOT) covered by one package of the drug** |
| LABA | salmeterol | SEREVENT DISKUS 50 MCG/DOSE | 30 |
| LABA | salmeterol | SEREVENT EVOHALER 25 MCG/DOSE | 30 |
| LABA | formoterol | ATIMOS 12 MCG/DOSE | 50 |
| LABA | formoterol | ATIMOS 12 MCG/DOSE | 60 |
| LABA | formoterol | FORADIL 12 MCG/DOSE | 30 |
| LABA | formoterol | FORMOTEROL EASYHALER 12 MCG/DOSE | 60 |
| LABA | formoterol | FORTOFAN 12 MCG/DOSE | 30 |
| LABA | formoterol | INHAFORT 12 MCG/DOSE | 90 |
| LABA | formoterol | INHAFORT 12 MCG/DOSE | 60 |
| LABA | formoterol | INHAFORT 12 MCG/DOSE | 60 |
| LABA | formoterol | INHAFORT 12 MCG/DOSE | 90 |
| LABA | formoterol | REVIFORM AXAHALER 12 MCG/DOSE | 30 |
| LABA | indacaterol | ONBREZ BREEZHALER 150 MCG/DOSE | 30 |
| LABA | indacaterol | ONBREZ BREEZHALER 300 MCG/DOSE | 30 |
| LABA | olodaterol | STRIVERDI RESPIMAT 2.5 MCG/DOSE | 30 |
| ICS/LABA fix | salmeterol and fluticasone | AIRFLUSOL SPRAYHALER 25 MCG/125 MCG/DOSE | 30 |
| ICS/LABA fix | salmeterol and fluticasone | DIMENIO 50 MCG/500 MCG/DOSE | 30 |
| ICS/LABA fix | salmeterol and fluticasone | FLUZALTO AIRMASTER 50 MCG/500 MCG/DOSE | 30 |
| ICS/LABA fix | salmeterol and fluticasone | FULLHALE 25 MCG/250 MCG/DOSE | 30 |
| ICS/LABA fix | salmeterol and fluticasone | SAFUMIX EASYHALER 50 MCG/500 MCG/DOSE | 30 |
| ICS/LABA fix | salmeterol and fluticasone | SALMETEROL/FLUTICASONE PROPIONATE PHAROS 50 MCG/500 MCG/DOSE | 30 |
| ICS/LABA fix | salmeterol and fluticasone | SERETIDE DISKUS 50/500 MCG/DOSE | 30 |
| ICS/LABA fix | salmeterol and fluticasone | SERETIDE EVOHALER 25/50 MCG/DOSE | 30 |
| ICS/LABA fix | salmeterol and fluticasone | THOREUS DISKUS 50/500 MCG/DOSE | 30 |
| ICS/LABA fix | formoterol and budesonide | BUFOMIX EASYHALER 4.5 MCG/160 MCG/DOSE | 30 |
| ICS/LABA fix | formoterol and budesonide | BUFOMIX EASYHALER 9 MCG/320 MCG/DOSE | 30 |
| ICS/LABA fix | formoterol and budesonide | DUORESP SPIROMAX 160 MCG/4.5 MCG/DOSE | 30 |
| ICS/LABA fix | formoterol and budesonide | DUORESP SPIROMAX 320 MCG/DOSE/9 MCG/DOSE | 30 |
| ICS/LABA fix | formoterol and budesonide | PULMALIO 200 MCG/DOSE/6 MCG/DOSE | 30 |
| ICS/LABA fix | formoterol and budesonide | PULMALIO 400 MCG/DOSE/12 MCG/DOSE | 30 |
| ICS/LABA fix | formoterol and budesonide | SYMBICORT 4.5 MCG/DOSE/160 MCG/DOSE | 30 |
| ICS/LABA fix | formoterol and budesonide | SYMBICORT FORTE TURBUHALER 9 MCG/320 MCG/DOSE | 30 |
| ICS/LABA fix | formoterol and budesonide | SYMBICORT TURBUHALER 4.5 MCG/DOSE/160 MCG/DOSE | 30 |
| ICS/LABA fix | formoterol and beclometasone | FOSTER 100 MCG/DOSE/6 MCG/DOSE | 30 |
| ICS/LABA fix | formoterol and beclometasone | FOSTER 100 MCG/DOSE/6 MCG/DOSE | 45 |
| ICS/LABA fix | formoterol and beclometasone | FOSTER NEXTHALER 100 MCG/DOSE/6 MCG/DOSE | 30 |
| ICS/LABA fix | formoterol and beclometasone | FOSTER NEXTHALER 100 MCG/DOSE/6 MCG/DOSE | 45 |
| ICS/LABA fix | vilanterol and fluticasone furoate | RELVAR ELLIPTA 92 MCG/DOSE/22 MCG/DOSE | 30 |
| ICS/LABA fix | salmeterol and budezonid | REVIX AXAHALER 120 MCG/DOSE/20 MCG/DOSE | 30 |
| LABA/LAMA fix | vilanterol and umeclidinium bromide | ANORO ELLIPTA 55 MCG/DOSE/22 MCG/DOSE | 30 |
| LABA/LAMA fix | indacaterol and glycopyrronium bromide | ULTIBRO BREEZHALER 85 MCG/DOSE/43 MCG/DOSE | 30 |
| LABA/LAMA fix | aklidinium-bromide and formoterol | BRIMICA GENUAIR 340 MCG/DOSE/12 MCG/DOSE | 30 |
| LABA/LAMA fix | olodaterol and tiotropium | SPIOLTO RESPIMAT 2.5 MCG/DOSE/2.5 MCG/DOSE. | 30 |
| ICS/LABA/LAMA fix | vilanterol, umeclidinium bromide and fluticasone furoate | TRELEGY ELLIPTA 92 MCG/DOSE/55 MCG/DOSE/22 MCG/DOSE | 30 |
| ICS/LABA/LAMA fix | formoterol, glycopyrronium bromide and beclometasone | TRIMBOW 87 MCG/DOSE/5 MCG/DOSE/9 MCG/DOSE | 45 |
| LAMA | tiotropium-bromide | BRALTUS 10 MCG/DOSE | 30 |
| LAMA | tiotropium-bromide | SPIRIVA 18 MCG/DOSE | 30 |
| LAMA | tiotropium-bromide | SPIRIVA RESPIMAT 2.5 MCG/DOSE | 30 |
| LAMA | aclidinium | BRETARIS GENUAIR 322 MCG/DOSE | 30 |
| LAMA | glycopyrronium bromide | SEEBRI BREEZHALER 44 MCG/DOSE | 30 |
| LAMA | umeclidinium bromide | INCRUSE ELLIPTA 55 MCG/DOSE | 30 |
| ICS/LABA/LAMA fix | formoterol, glycopyrronium bromide and beclometasone | TRIMBOW 88 MCG/DOSE/5 MCG/DOSE/9 MCG/DOSE | 30 |

| **Table 2. Total prices (HUF) of the maintenance inhaled drugs with the highest market shares in Hungary between 2011 and 2019** | | | | | | | | | |
| --- | --- | --- | --- | --- | --- | --- | --- | --- | --- |
|  | 2011 | 2012 | 2013 | 2014 | 2015 | 2016 | 2017 | 2018 | 2019 |
| FOSTER pMDI 100 μg/ 6 μg | 15 303 | 15 367 | 15 367 | 13 935 | 13 549 | 12 783 | 12 783 | 12 783 | 12 783 |
| SPIRIVA HANDIHALER 18 μg | NA | 11 120 | 11 120 | 11 120 | 11 120 | 11 120 | 11 120 | 11 120 | 8 863 |
| SPIRIVA RESPIMAT 2,5 μg | NA | 11 120 | 11 120 | 11 120 | 11 120 | 11 120 | 11 120 | 11 120 | 11 120 |
| SYMBICORT TURBUHALER 4,5 μg/160 μg | 13 579 | 13 653 | 13 653 | 11 131 | 10 147 | 9 004 | 9 004 | 9 004 | 9 004 |
| ULTIBRO BREEZHALER 85 μg/43 μg | NA | NA | NA | NA | 16 591 | 16 591 | 16 591 | 16 591 | 16 591 |
| HUF: Hungarian Forint, NA: not available in Hungary in that calendar year | | | | | | | | | |

| **Table 3. ICD codes and VIF for individual comorbidities** | | |
| --- | --- | --- |
| Individual comorbidity included in the model | International Statistical Classification of Diseases and Related Health Problems 10th Revision (ICD-10) | VIF |
| Acute myocardial infarction | I21 | 1.068751616 |
| Anaemia | D50-D53, D55-D64 | 1.034723141 |
| Anxiety | F40-43 | 1.045229616 |
| Asthma | J45 | 1.024847372 |
| Atrial fibrillation and flutter | I48 | 1.071084957 |
| Cancer | C00-C76, C80-C97 | 1.614526599 |
| Cerebrovascular diseases | G45-G46, I60-I69 | 4.366433972 |
| Cerebrovascular stroke | I60-I69 | 4.339769592 |
| Congestive heart failure | I099, I110, I130, I132, I255, I420, I425-I429, I43, I50, P290 | 1.109509239 |
| Connective tissue disorder | M05-M06, M315, M32-M34, M351, M353, M36 | 1.007533029 |
| Dementia | F00-F04, F051, G30, G311 | 1.013943114 |
| Depression | F31-34 | 1.029095228 |
| Diabetes | E10-14, M14, N08 | 1.164134428 |
| Diabetic complications | E102-E105, E107, E112-E115, E117, E122-E125, E127, E132-E135, E137, E142-E145, E147 | 1.139024505 |
| Hypertension | I10-15, I67 | 1.098985732 |
| Ischemic heart diseases | I20-25 | 1.167677212 |
| Liver disease | B18, K700-K717, K73-K74, K76, Z944 | 1.005445392 |
| Lung cancer | C34 | 1.600050178 |
| Metastatic cancer | C77-C80 | 1.093350145 |
| Osteoporosis | M80-82 | 1.052310476 |
| Paraplegia | G041, G114, G801, G802, G81-G82, G830-G834, G839 | 1.006889935 |
| Peptic ulcer | K25-K28 | 1.008347355 |
| Peripheral vascular disease | I70-I71, I731, I738, I739, I771, I790, I792, K551, K558, K559, R02, Z958, Z959 | 1.025879615 |
| Other pulmonary disease | I278, I279, J40-J43, J46-47, J60-J67, J684, J701, J703 | 1.014078316 |
| Renal disease | I12, I13, N01, N03, N052-N057, N072-N074, N18, N19, N25, Z490-Z492, Z992 | 8.496715393 |
| Renal failure | N18-19 | 8.488893282 |
| Sleep apnoea | G4730 | 1.004138383 |

| **Table 4. AIC values from different models in sensitivity analysis** | | | | |
| --- | --- | --- | --- | --- |
|  | All-cause total cost | All-cause inpatient cost | COPD-specific total cost | COPD-specific inpatient cost |
| Original model | 1 370 963 | 561 377 | 1 119 811 | 151 013 |
| Model with individual comorbidities | 1 363 029 | 559 666 | 1 119 010 | 150 931 |
| Model without adjustment to maintenance inhaled therapy days | 1 373 959 | 561 658 | 1 172 853 | 151 010 |
| Model without adjustment to hospitalization number | 1 382 886 | 565 590 | 1 182 719 | 155 576 |
| Model without adjustment to maintenance inhaled therapy days and hospitalization number | 1 385 307 | 565 817 | 1 208 663 | 155 604 |

| **Figure 1. The effects of hospitalization frequency, age and therapy-covered days on total all-cause healthcare costs** | | |
| --- | --- | --- |
| 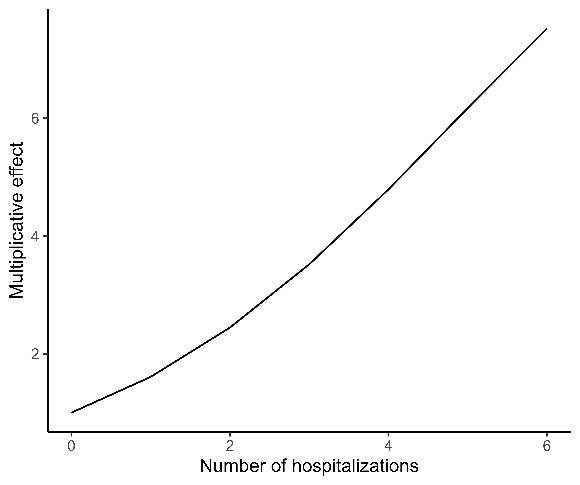 | 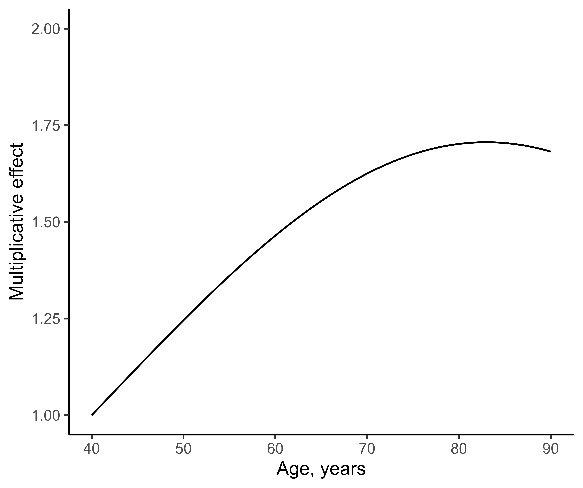 | 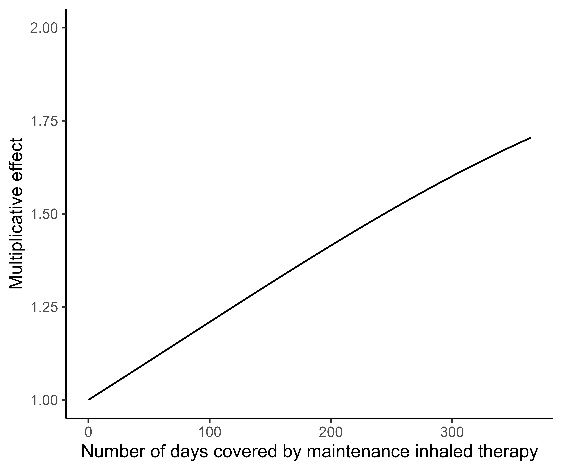 |

| **Figure 2. The effects of hospitalization frequency, age and therapy-covered days on the costs of all-cause hospitalizations** | | |
| --- | --- | --- |
| 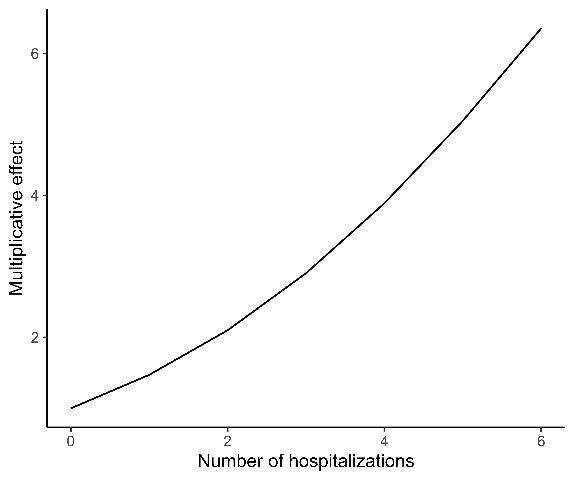 | 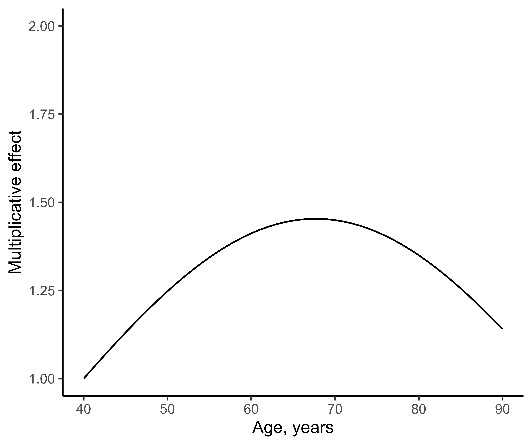 | 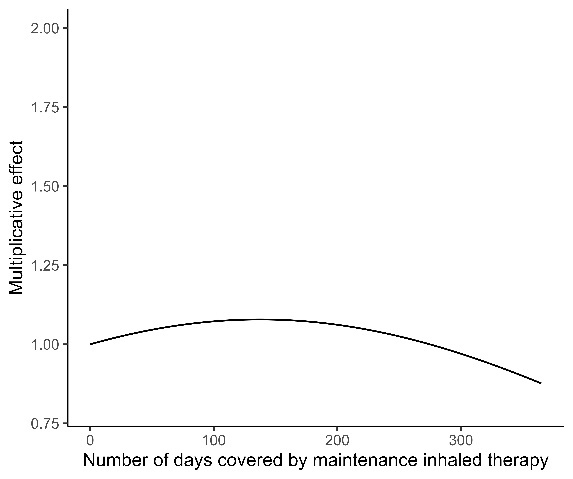 |

| **Figure 3. The effects of hospitalization frequency, age and therapy-covered days on total COPD-related healthcare costs** | | |
| --- | --- | --- |
| 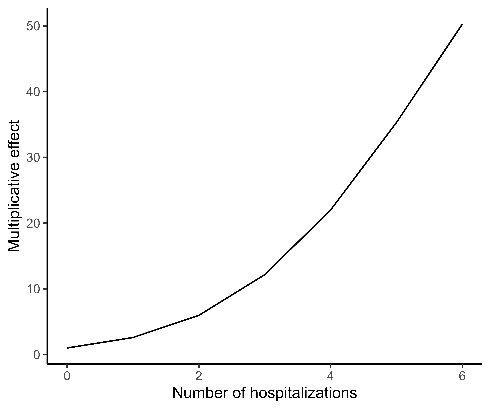 | 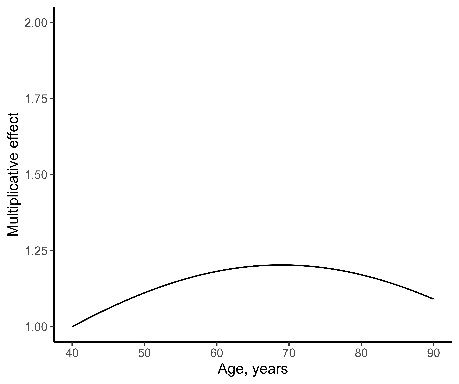 | 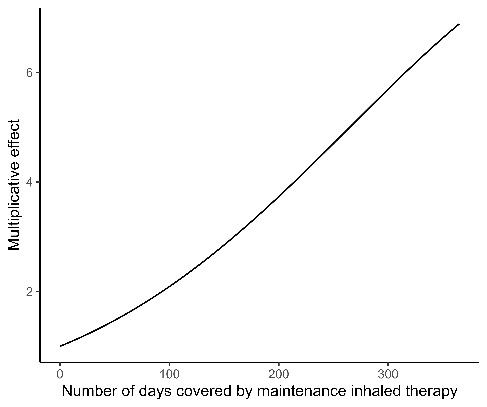 |

| **Figure 4. The effects of hospitalization frequency, age and therapy-covered days on the costs of COPD-related hospitalizations** | | |
| --- | --- | --- |
| 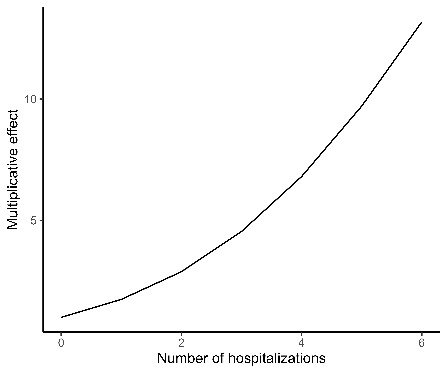 | 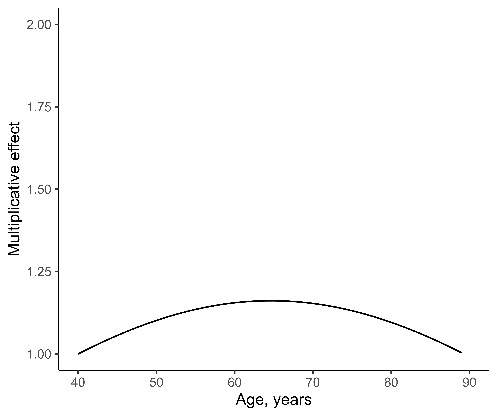 | 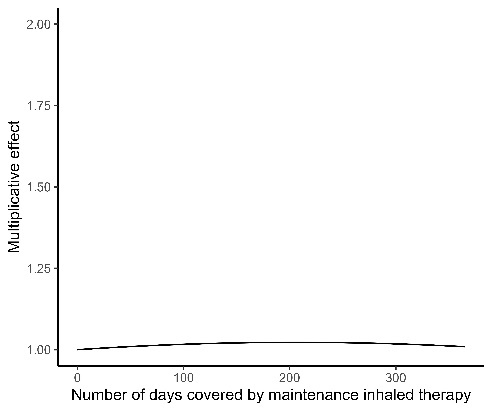 |

| **Figure 5. Model diagnostics for all-cause total, all-cause hospitalizations, COPD-related total and COPD-related hospitalization costs** | |
| --- | --- |
| **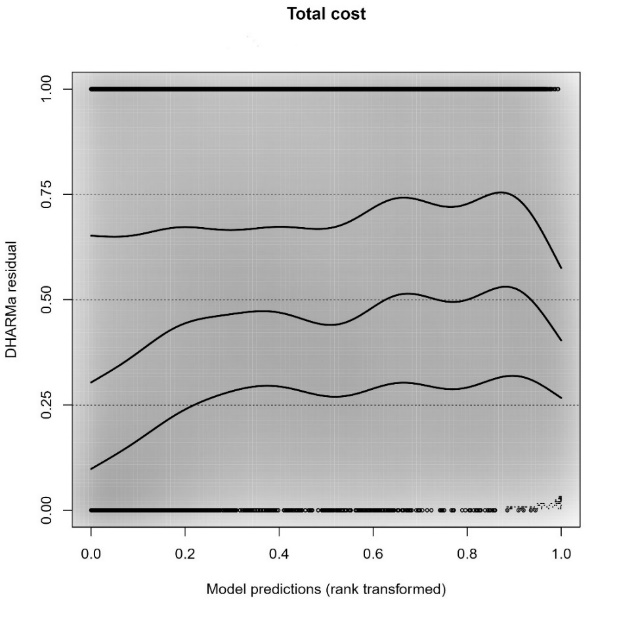** | 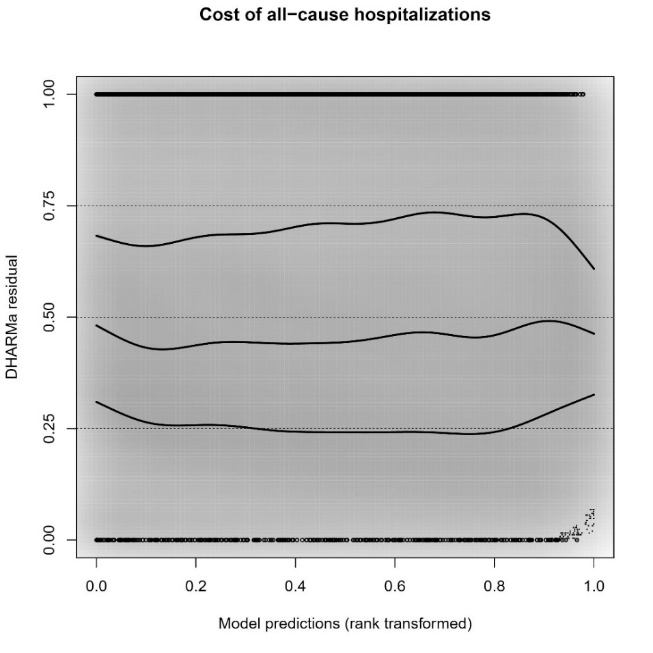 |
| 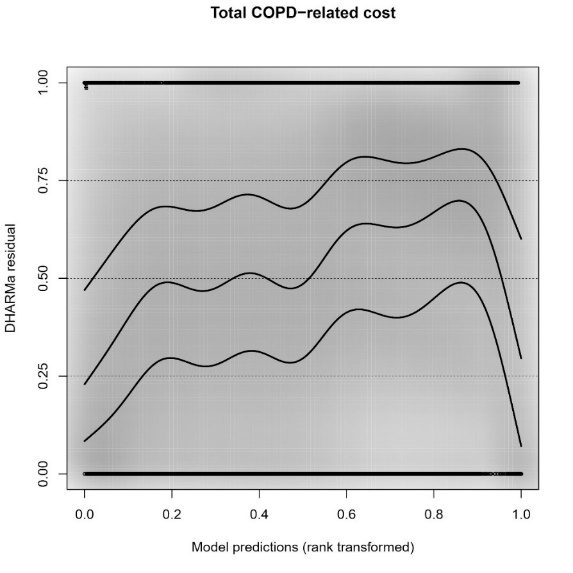 | 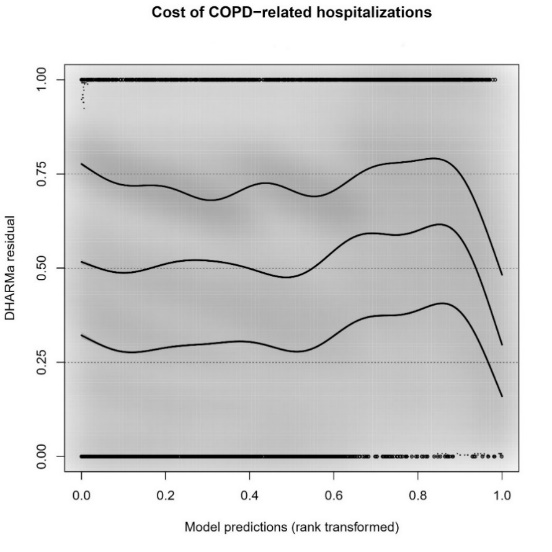 |

**Figure 6. The association of clinical variables and individual comorbidities to all-cause total healthcare costs.**

**
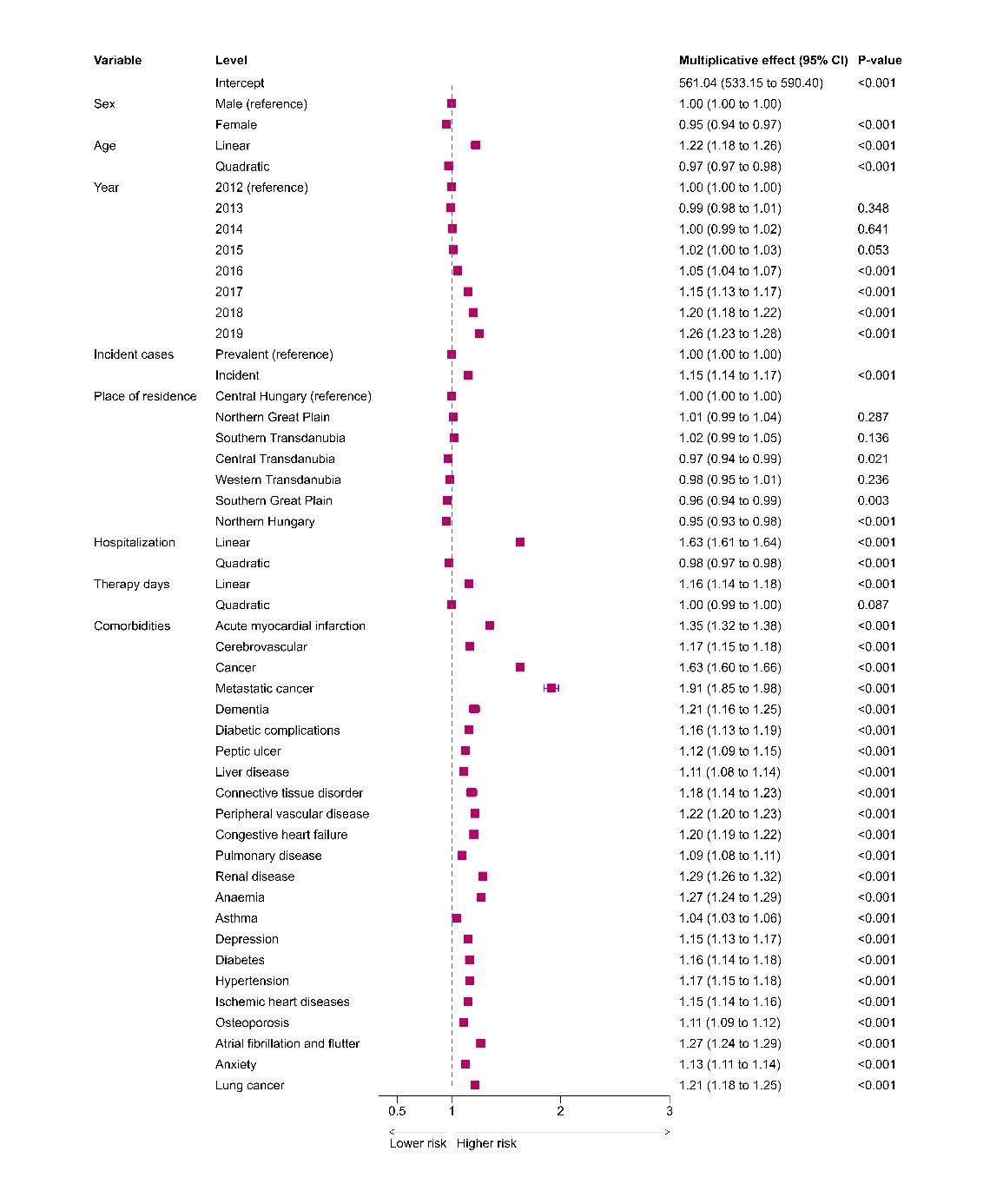
**

**Figure 7. The association of clinical variables and individual comorbidities to all-cause inpatient healthcare costs.**

**
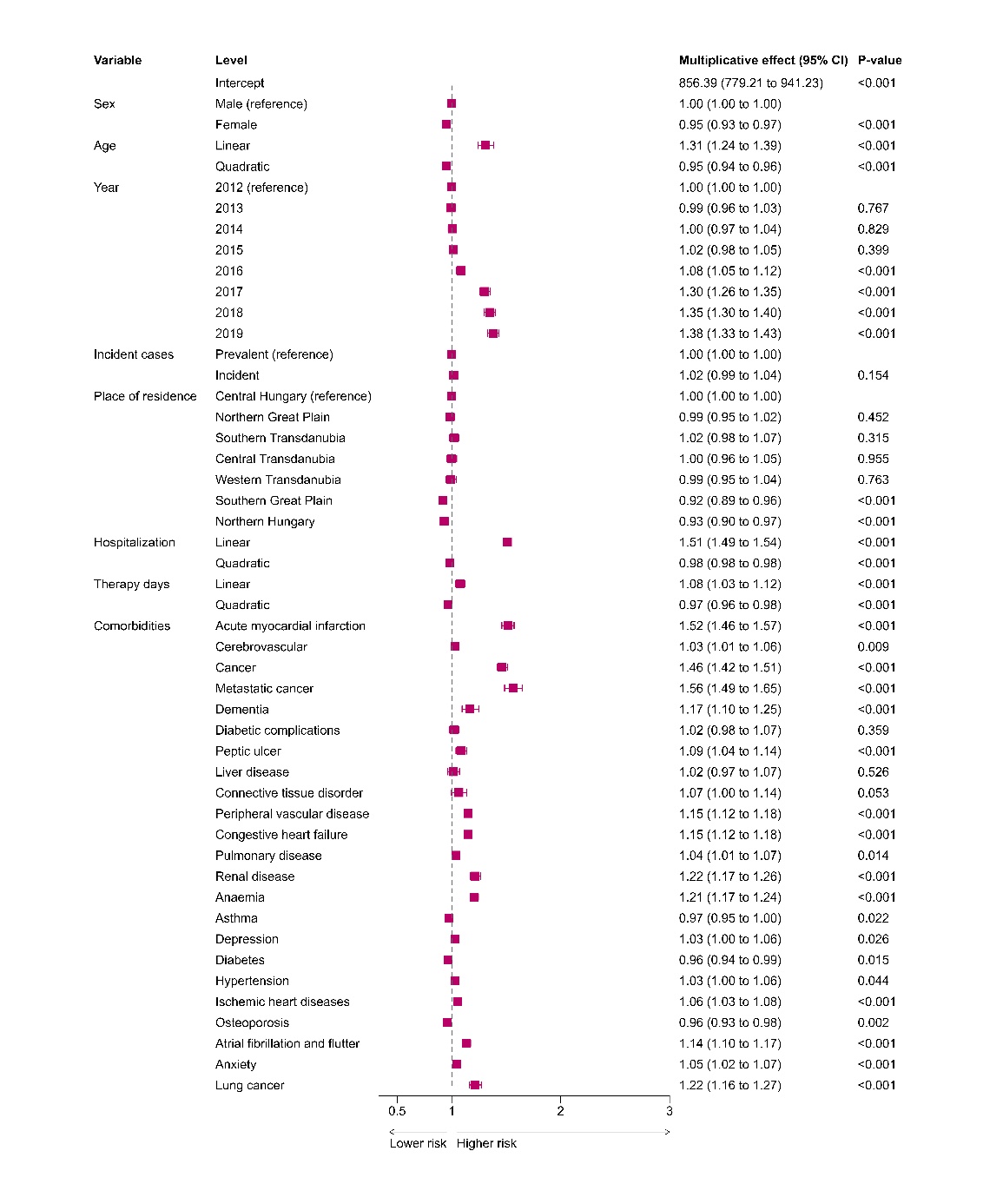
**

**Figure 8. The association of clinical variables and individual comorbidities to COPD-specific total healthcare costs.**

**
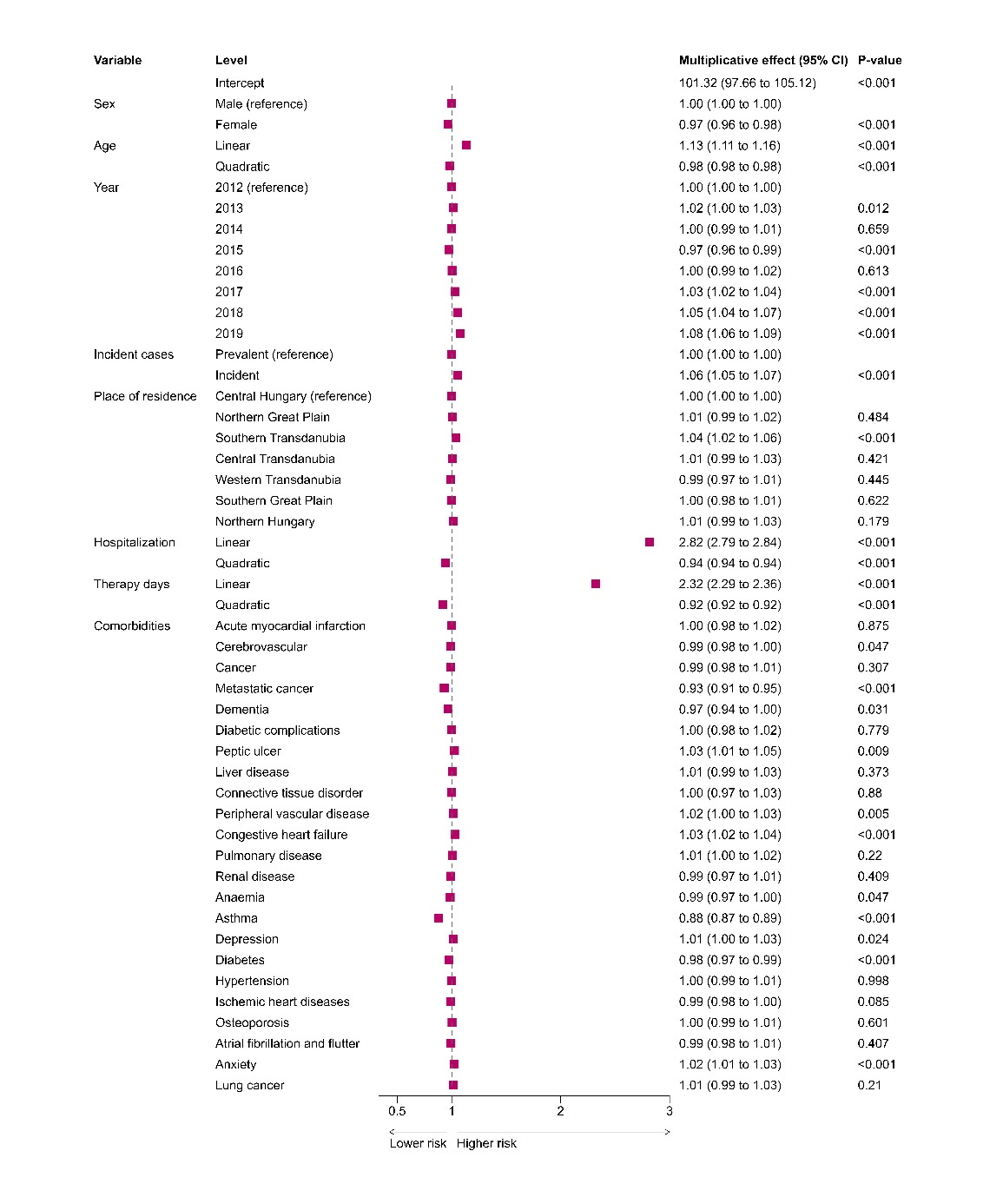
**

**Figure 9. The association of clinical variables and individual comorbidities to COPD-specific inpatient healthcare costs.**

**
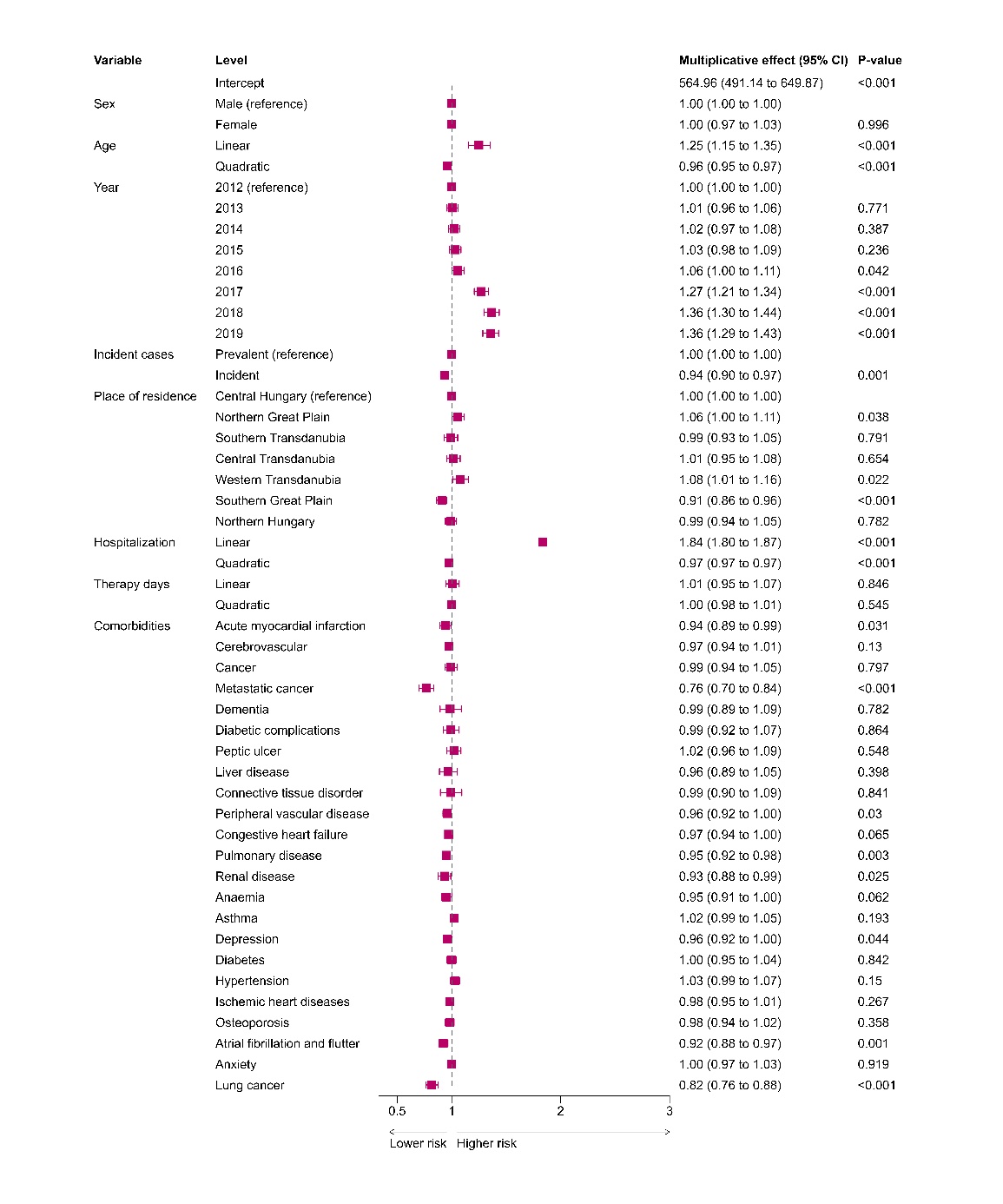
**
