# Supplementary material for: Comparison of healthcare costs of patients with COPD on maintenance inhaled therapies between 2011 and 2019 in Hungary using a nationwide database
Source: PLoS One. 2025 May 5;20(5):e0320949. doi: 10.1371/journal.pone.0320949 (PMC12052116; doi:10.1371/journal.pone.0320949)
Supplement: Attachment — Submitted filename: Response_to_reviewers_final.docx [file pone.0320949.s003.docx]

Dear Prof. Kuo-Cherh Huang,

Thank you for the opportunity to review our manuscript. We assessed all points raised by the reviewers and responded to all comments in a point-by-point manner, please see below.

**Reviewer #1: PONE-D-24-3126**

Comparison of healthcare costs of patients with COPD on maintenance inhaled therapies between 2011 and 2019 in Hungary using a nationwide database

**Comment: Keywords: Financial Database (not a mesh term)**

Response: Thank you, we reviewed the term “Financial Database” with “Database Management Systems”, which is listed as a MeSH term.

Introduction:

**Comment: Line 59: COPD also poses a major economic burden on high income countries and their healthcare systems [1]: Please adjust the reference to be more specific. And be a little clearer regarding the economic burden on health systems, regarding the use of resources in COPD. In general: Please be a little more specific regarding the reported economic burden: Is it more in medicines? What proportion? Or in clinical care? Or in oxygen?**

Response: Thank you for the observation, we restructured this paragraph in the Introduction to more specifically report the findings of previous studies. We also added a new reference (Foo et al.  Continuing to Confront COPD International Patient Survey: Economic Impact of COPD in 12 Countries), which compared data on direct costs of COPD, including those on home oxygen treatment, in 12 countries using data from surveys.

Methods:

**Comment: Please clarify who are the candidates to be insured by the public NHIF system, for readers who are not familiar with the Hungarian health system. – Is it the entire population of the country? The unemployed? Those without income?**

Response: NHIF covers all Hungarian citizens who pay health insurance, which is generally automatically deducted from monthly salaries, so all people who are employed in Hungary are automatically eligible for insurance. Also, all people under 16 years of age, and all full-time students (including high school, university or other educational institutions) below or above 18 years of age, all pensioners are insured. Finally, unemployed people are also eligible to apply for insurance for free.  Based on this we added further details to the Methods (lines 88-97) and the Supporting Information.

**Comment: Insurance number: Is it a unique national identification number? Is it used for other daily aspects? Or only health insurance?**

Response: Thank you for the insightful comment. Insurance numbers or social security numbers are unique identifiers for all people in Hungary, provided at birth. This identifier is only used to access the healthcare data and health insurance status (eligible for treatment or not, based on insurance paid) of everyone, it is not applicable for unique identification of a person. We specified this aspect in the revised manuscript (lines 86-98).

Patients:

**Comment: Line 102: Age over 40 or ≥ 40 years. Please clarify.**

Response: We collected data from patients older than 40 years of age, this was also corrected on Figure 1 as exclusion ≤ 40 years of age.

**Comment: Line 103: Medications: It is worth clarifying which therapies were included, perhaps in a supplementary table. How were they linked to the DB? A unique code for the medications? Were short-acting bronchodilators also included? Were other therapies such as LABA/LAMA included? – ICS alone? Roflumilast? Etc?**

Response: The exact type of medication (drug and brand names) is recorded on the prescriptions which are linked to the patients’ insurance number. If a patient fills that prescription in, the medication is linked to the patient within the NHIF database. Only maintenance treatment of COPD was assessed in the present study, meaning that short-acting bronchodilators were not included. As discussed in the first paragraph of the Results and in Table 1, LABA and LAMA monotherapies, combinations and their combination with ICS were also included. ICS and roflumilast monotherapies are not recommended as maintenance options for COPD treatment, hence they were not studied. Also, roflumilast is not available in Hungary, therefore it was totally excluded from the study. We added a new table (S1 Table) to the Supporting information listing all drugs and their brand names which were available as inhaled maintenance therapy for J41-44 ICD-10 codes in Hungary between 2011 and 2019.

Data collection:

**Comment: Line 115: Is CCI calculated by default in the database record? Or was it calculated by the authors?**

Response: CCI was calculated by the authors, based on the methodology by Quan et al. (Quan H, Sundararajan V, Halfon P, Fong A, Burnand B, Luthi JC, Saunders LD, Beck CA, Feasby TE, Ghali WA. Coding algorithms for defining comorbidities in ICD-9-CM and ICD-10 administrative data. Med Care. 2005 Nov;43(11):1130-9. doi: 10.1097/01.mlr.0000182534.19832.83. PMID: 16224307). This was clarified in the revised manuscript, and the reference was added (lines 139-40).

**Comment: Line 117: Medical costs reimbursed by the NHIF? What costs are not covered by the system – Worth clarifying.**

Response: We fully agree with the reviewer. Healthcare costs are fully or partially covered by NHIF. All costs of out- and inpatient care, laboratory tests, imaging examinations are fully reimbursed, while certain percentage of costs of medication (in case of COPD inhaled maintenance medications - 90%, oxygen therapy – 100%) and medical aids (usually 50%) are covered. If medication is fully reimbursed by the NHIF, the patient still has to pay 300 HUF (0.77 USD as of Nov 2024) for filling in a prescription. This is detailed in the Supporting information of the revised manuscript.

**Comment: Line 124: All costs in 2011 dollars? Why not all with 2019 costs and a discount rate applied? Otherwise the costs would be estimated without taking inflation into account for almost a decade.**

Response: During the 2011-2019 period, inflation and fluctuations in the HUF-USD exchange rates could have influenced overall costs. In our analysis, we took multiple financial factors into account.

Firstly, we should note that the annual inflation rate in Hungary was relatively low during this period, with an average rate of 2.22%, and a cumulative rate of 23.1% between 2011 and 2019, as also discussed in the original and in the revised manuscript (lines 153-6). The highest inflation occurred in 2012 at 5.7%, which we used as the reference year in our model. Additionally, many basic therapeutical costs were price-controlled by the National Health Insurance Fund (NHIF), and these rates were not adjusted to the rate of inflation. Specifically, here we show that there was no increase in the total prices of the most frequently prescribed maintenance inhaled medications for COPD during the study period using publicly available information. Indeed, due to the appearance of generic drugs, the price of some drugs have declines (e.g. the LAMA tiotropium, which used to be marketed as the patented Spiriva Handihaler, but after the expiration of the patent the drug is now available in various formats of dry powder inhalers, leading to a decrease in the price of the original medication).

However, the standardized amount per Diagnosis Related Groups reimbursed by the NHIF increased in the study period.

Secondly, the HUF-USD exchange rate increased by over 50% between 2011 and 2019. If we had recalculated the USD value annually, it would have – misleadingly - shown a significant decrease in costs, largely due to the strengthening of the USD currency.

However, to avoid interpretation bias we did not adjust the costs to inflation or currency exchange rates. Considering these factors, we decided to present the original cost values without adjusting for inflation. To provide context for readers, we converted these values to USD, using a fixed exchange rate. However, we mention this as a limitation of the study in the revised manuscript (lines 353-7).

Statistical analysis:

**Comment: For the linear regression model: The days covered by inhaled therapy were included as covariates, however, it is not clear whether everything was included in general as therapy or by group of medication or by molecular used. – Please clarify, because this is a factor clearly related to costs.**

Response: Days covered by inhaled therapy was a covariate, that measures the number of days the prescribed drug provided inhaled therapy for. We constructed a therapy vector from each reimbursed medication, based on the days of treatment (DOT) covered by the drug package (please see S1 Table in the revised manuscript), and interpreted it as the number of covered days. This was used as one covariant (scaled by 100 days).

Results:

**Comment Line 149: They talk about lack of adherence. How can we be sure that it is a lack of adherence? Or could it be a bad diagnostic classification? Or failures in the delivery and access to therapy? Adverse reactions? Suspension of therapy by the treating physician?**

Response: We agree with the reviewer that the sentence of “The annual number of patients increased in the study period, but this rise was lower than the corresponding number of incidence cases in that year, showing a lack of adherence to inhaled maintenance therapies” raises concerns. We have reassessed this issue and acknowledge that lack of adherence is not an explanation for this phenomenon, as all patients who met the inclusion criteria of 3 months of inhaled maintenance therapy are consequently included in the study population irrespective of future adherence. However, those patients who are included but did not have medical costs in the given year were excluded from the cases. Importantly, the number of these cases was negligible as shown in the table below:

|  | **2012** | **2013** | **2014** | **2015** | **2016** | **2017** | **2018** | **2019** |
| --- | --- | --- | --- | --- | --- | --- | --- | --- |
| **Number of patients with zero costs** | **<10** | **<10** | **<10** | **16** | **16** | **15** | **14** | **11** |

Thus, the explanation for the increase in the study population being lower than the annual number of incident case is the mortality rate, we have rewritten the sentence in the Results. This was also mentioned in the Methods as “Further data were collected for each calendar year for the enrolled patients if the patients were alive on 1 January in the same year.” (lines 131-2).

**Comment: It is interesting that an incremental cost is found in hospital and outpatient care. Although I do not see the number of hospitalizations in the results. It is worth including it. Is it due to a greater number of hospitalizations per patient? Or is it the same average but with an increase in costs. Please clarify, and for these reasons it is important to take into account price inflation for the analysis.**

Response: Inpatient and outpatient expenditures are costs of all events per person in that certain year. We have added and extra row to the descriptive statistics table (Table 1) that shows the average hospitalizations of a person over the years to COPD. It seems that the 0.19 (sd 0.69) as the mean yearly number of hospitalizations did not change significantly over the years, therefore the cost increment should be explained by other factors (e.g. longer hospitalizations, increased costs, more treatments or more advanced therapies at the hospital). This was also added to the Discussion (lines 307-13).

**Comment: When looking at the figures, they are of low quality and very pixelated. They cannot be evaluated. Send in better resolution. To review correctly.**

Response: We improved the quality of the figures.

Discussion:

**Comment: Include in the discussion why the increase in costs related to hospitalization? More or higher costs of these? And the potential reasons for this increase. Keeping in mind that, in theory, the main impact of current therapies is related to the reduction of the number of exacerbations. Or definitely patients are not continuously accessing therapies and it is a problem to be further explored in the health system. Expand strengths and limitations.**

Response: Thank you for the comment. As shown above, the annual number of hospitalizations/patients because of COPD was relatively stable in the study period. Several factors can explain the link between the number of hospitalizations due to COPD and costs. Firstly, in-patient treatment in general costs more than outpatient care. The duration of hospital treatment and interventions during hospital stay also influence related healthcare costs. Specific to COPD, in the last 10 years, access to non-invasive ventilation as a treatment modality of COPD exacerbations with respiratory failure has greatly improved in Hungary with higher daily costs of therapy, possibly also contributing to higher overall costs (lines 307-13).

We fully agree with the reviewer that one of the main aims of the medical treatment of COPD is to prevent exacerbations. We also acknowledge that access to healthcare (both medical care and medication) is not homogenous in any countries which is also supported by our findings that certain regions in Hungary are associated with higher or lower costs of hospital care (both all-cause and COPD-related). Importantly, several factors have been described to influence COPD care, and thus maybe also costs, including lack of patient education, availability of social support, distance to health facilities, and attitude of healthcare providers (Nguyen TA et al. BMC Public Health 2021 DOI: 10.1186/s12889-021-11219-4), but perceptions of ease of access, quality of relationship with their general practitioner or perceived disease severity can also play a role (Shipman C et al. Prim Care Respir J 2009 DOI: 10.4104/pcrj.2009.00013). Unfortunately, we were not able to explore the influence of these factors on the costs, as our database could not be enriched by these additional parameters from the sources of the NHIF. We discuss these issues in the limitation of our study, and added the two references (lines 358-65).

**Reviewer #2: In this study, authors examined the temporal trends in the costs of COPD in a Hungarian population from 2011 to 2019. While the findings are interesting, I do have the following suggestions, Comments and concerns:**

**Comment1. Not familiar with the phrase ‘which corresponds with a decline in real value for the latter’**

Response: Thank you for the comment. We wanted to highlight that this increase in costs was less than the corresponding inflation rate, so we rewrote this sentence in the abstract.

**Comment2. Would recommend addition temporal findings in abstract (if space constraints, some of the methods can be concatenated)**

Response: Thank you for the valuable comment. The abstract was rewritten, and temporal findings were included.

**Comment3. Intro: International Classification of Diseases (IDC-10) should be ICD-10**

Response: We apologize for the typing mistake, we corrected it.

**Comment 4. Methods: Rationale for 40y/o+ can be added for readers not familiar with the disease**

Response: COPD usually develops over decades, mainly due to repeated exposure of harmful effects (e.g.: cigarette smoking, biomass burning, repeated pulmonary infections etc.). Hence, exclusion of younger patients can help reduce the number of misdiagnosis or at least, misclassified patients. Misclassification here means the incorrect use of ICD-10 codes due to administrative error. It has recently been shown in a population-based study focusing on patients < 50 years with early COPD, that almost 90% of patients in that age group were > 40 years old (Am J Respir Crit Care Med 2020 Mar 15;201(6):671–680). We added this information to the revised manuscript (line 121).

**Comment 5. Methods: how were patients with co-existing asthma handled? Since it is a common comorbidity- it should be included and adjusted for**

Response: Patients with concomitant asthma and COPD (COPD-A) were excluded, by limiting enrolment to patients who had most of their healthcare appearances with J44 code as compared to J45. A strict, absolute exclusion of the J45 code would limit our population too much, considering that it would exclude patients with misclassifications and those with difficult differential diagnosis especially at the beginning of the disease course. Therefore, to ensure exclusion of earlier misdiagnosed bronchial asthma, patients were excluded if more than 20% of their last 10 prescriptions were registered with J45 ICD-10 code, as stated in the original manuscript (lines 129-31 in the reviewed manuscript).

**Comment6. Methods: Would recommend including patients for duration of follow-up once diagnosed, even if zero costs. This is due to the irreversible nature of COPD and potential to overestimate costs due to exclusion of milder cases. Can be a supplemental analysis too.**

Response: Patients with zero costs cannot be followed in this database, considering that their cost can only be zero, if they do not use any healthcare resource each year, which means that they will not be present in the database. In addition, we aimed to assess the healthcare utilization of patients on treatment, which by itself would generate cost.

In this patient group, it is very rare to observe zero costs in a year. In fact, fewer than 20 patients had zero costs each year, which can be considered not relevant compared to the total patient population in our analyses. Furthermore, zero costs are not allowed in the Gamma regression model, meaning that a more complex zero-inflated Gamma model would be required. However, given the very low number of zero-cost patients, such a model would likely produce estimates with high variance. Therefore, we believe that including these patients in the model would not significantly improve its accuracy or data interpretations, but it would rather introduce large variances. The number of patients with zero costs are listed below in the table (note that subgroups with fewer than 10 patients cannot be presented due to NHIF regulations). This was clarified in the revised manuscript (lines 145-6).

|  | **2012** | **2013** | **2014** | **2015** | **2016** | **2017** | **2018** | **2019** |
| --- | --- | --- | --- | --- | --- | --- | --- | --- |
| **Number of patients with zero healthcare costs** | <10 | <10 | <10 | 16 | 16 | 15 | 14 | 11 |

**Comment7. Not sure I followed how every patient in 2011 is an incident patient. Would the prevalent users not be captured?**

Response: We considered patients incident in a calendar year if they met the inclusion criteria first time i.e. received COPD maintenance inhaled therapy i.e. long-acting muscarinic antagonists (LAMA) or long-acting beta2-agonists (LABA) alone or in combination, or together with inhaled corticosteroids (ICS), prescribed with J41-44 ICD-10 codes. Since the dataset was available starting in 2011, we were unable to determine a patient’s incident or prevalent status for that year. Overcoming this bias, we analysed the data only from 2012 onwards, when we could establish the patient status based on data from the previous year. We added this definition to the revised manuscript (lines 146-9). While there could be some bias in later years if a patient was previously prevalent but had a therapy gap throughout 2011, it has a low likelihood as COPD is a progressive disease with high symptom burden. However, we added this as a limitation of our study (lines 333-8).

**Comment8. I do not think CCI can fully capture the comorbidity burden and recommend using individual comorbidities instead.**

Response: We fully agree with the reviewer that the use of CCI reduces the number of comorbidities that can be evaluated in the model. However, inclusion of all comorbidities in our analysis would not feasible, as their high number would reduce the intelligibility of the model. So, a selection of comorbidities is necessary. CCI is a well-known standardized predictor of mortality through all patient groups, and it could give valuable clinical associations to the outcomes of healthcare costs. However, to support that the set of comorbidities in in our study population is similar to other cohorts,  here we list the 15 most common accompanying diseases in a cohort which was generated from the same database, and recently published by our group (Vincze K et al. Orv Het 2024, doi: 10.1556/650.2024.32981 - fulltext only available in Hungarian): hypertension, ischemic heart disease, bronchial asthma, anxiety, heart failure, cerebrovascular events (stroke, transitory ischemic attack), diabetes, osteoporosis, depression, arrhythmia (flutter, atrial fibrillation), anaemia, lung cancer, renal failure, aorta aneurysm (data not shown).

In our study the main aim was to find out the effect of calendar years costs for a patient. We used the GLM Gamma regression method, as it is considered as one of the best for population means (Malehi, A.S., Pourmotahari, F. & Angali, K.A. Statistical models for the analysis of skewed healthcare cost data: a simulation study. Health Econ Rev 5, 11 (2015).). In addition to the calendar years, we included common, well-known cost-related factors as covariates (age, sex, comorbidity, hospitalization, therapy length etc.). Some of these factors could also be potential outcomes, but our primary goal was to adjust with these covariates to make the effect of years more accurate. We decided to use CCI over separated comorbidities for statistical reasons. Using individual comorbidities, the risk of multicollinearity is very high, as comorbidities often occur together. This can cause instability and make the interpretation more difficult (i.e. cardiovascular disease and diabetes). Moreover, using too many covariates can result in overfitting in the model.

**Comment: 9. Why is number of days of therapy included in the model? Wont adjusting for this variable lead to an overestimation of costs?**

Response: Thank you for the comment. Number of days of therapy represents the adherence of patients to treatment. It is true that it is directly proportional to medication costs, but it can also have a strong effect on COPD-related outcomes, or even outcomes of comorbidities.

Days of therapy and the number of hospitalizations is known to increase medical cost, as every therapy has a price. We included these variables as explanatory factors in our models to account for these cost variations over the years. The model coefficients suggest that these factors are truly the most relevant ones, but the calendar years were in the centre of our focus, therefore adjusting is necessary. With this covariate, we aimed to include adherence into the model. We had two options: (1) using the days covered by therapies, or (2) using the number of prescriptions. We decided to use the first option as the same number of prescriptions can result in different therapy length as the days of treatment (DOT) per drug packages can be different (see S1 Table in the Supporting Information).

**Comment10. Why is number of hospitalizations included in the model? This is an outcome variable (through hospitalization costs)- as demonstrated in findings.**

Response: Thank you for raising this issue. Acute severe exacerbations were defined as hospitalisations or emergency ward visits with the ICD-10 code of COPD. This definition is not just used in financial database research but is also in line with current COPD guidelines. The number of severe exacerbations can increase costs not just through the cost of the COPD-related hospitalisations, but through the need for more medication and medical aid use for stable COPD and more resource spent on the control of comorbidities (as it is proven that the risk of worsening of multiple comorbidities is higher after an exacerbation). Taken all into account, we considered the inclusion of exacerbations necessary in the model.

**Comment11. Based on the results shown in table 2, the costs are not statistically significantly more? There is a very high SD. Not sure that the conclusions of higher costs can be made based on these findings.**

Response:  We acknowledge the concern of the reviewer. Table 2 presents the descriptive statistics for average costs across years. Costs do not follow normal distribution but usually can be modeled well with some right-skewed distribution with large variances, such as Gamma ((Malehi, A.S., Pourmotahari, F. & Angali, K.A. Statistical models for the analysis of skewed healthcare cost data: a simulation study. Health Econ Rev 5, 11 (2015).). With these properties, one cannot make statistical conclusions just by solely using descriptive statistics as usual tests are designed for normal distributions. Therefore, table 2 suggests some increment in total inpatient and outpatient costs, but another adequate model was needed to assess statistical significance (or non-significance). This is confirmed in Figures 2 and 3, where that there was a significant increment over the years. Consequently, the statistical significance in costs over years could not be studied by the descriptive analysis (table 2), but it was analysed using the regression models (Figures 2,3). This was also detailed in the Methods of the revised manuscript (lines 161-3).

**Comment12. Figures 2 and 3 are very blurred- are they based on the regression coefficients? Is statistical significance considered.**

Response: Thak you for noticing it. Figures 2 and 3 are so called ‘forest plots’ (https://cran.r-project.org/web/packages/forestploter/vignettes/forestploter-intro.html), where the model coefficients and its confidence intervals are presented simultaneously on the same scale. P-values are also presented in the figure in the 5th column. As the standard deviations were extremely small, the confidence intervals seem like a single point. A significant difference compared to the base value (year 2012) can be considered when the p-value is less than 0.05, which is the case for almost every calendar year. The quality of the figures has been improved in the revised manuscript.
