# Supplementary material for: Comparison of healthcare costs of patients with COPD on maintenance inhaled therapies between 2011 and 2019 in Hungary using a nationwide database
Source: PLoS One. 2025 May 5;20(5):e0320949. doi: 10.1371/journal.pone.0320949 (PMC12052116; doi:10.1371/journal.pone.0320949)
Supplement: Attachment — Submitted filename: Response_reviewer2.docx [file pone.0320949.s004.docx]

Dear Prof. Kuo-Cherh Huang,

Thank you for the opportunity to review our manuscript. We assessed all points raised by the reviewer and responded to all comments in a point-by-point manner, please see below.

**Reviewer #2: The authors have addressed most comments, but the ones below still need work.**

**Comment:** For comment 8- VIF and tolerance can be tested for multicollinearity and knowing specific comorbidities and their association with costs is more valuable (of course higher CCI is related to higher costs- does not tell much). Specifically for comments 9 and 10, if the goal of the study is to estimate the costs, there is a need to reconsider the covariates as originally suggested or at the least, conduct sensitivity analysis- if authors still have access to the data. Alternatively, the focus can be just on factors affecting costs, and the trends piece/estimation can be removed as factors on the causal pathway for costs are included and adjusted for. If the authors do not have access to the data anymore- I understand that they cannot carry out additional analysis - but they do need to clearly and explicitly discuss implications of these choices in the manuscript.

**Response**: Thank you for the insightful comment.

We were able to access the original database, however, due to computational constraints, the additional sensitivity analysis could be carried out only on a representative subsample (20 000 patients, random sampling).

According to the suggestion of the reviewer, we collected the most relevant comorbidities also based on our previous publication (doi: 10.1556/650.2024.32981). In a single model, we aimed to determine their collinearities by VIF values. A total of 27 types of comorbidities were analysed (see below and also as S3 Table in the Supporting information). Rare comorbidities (< 1%) and comorbidities with high multicollinearity (> 4), measured by VIF were excluded from further analysis.

| **Individual comorbidities included in the model** | **International Statistical Classification of Diseases and Related Health Problems 10th Revision (ICD-10)** | **VIF** |
| --- | --- | --- |
| Acute myocardial infarction | I21 | 1.068751616 |
| Anaemia | D50-D53, D55-D64 | 1.034723141 |
| Anxiety | F40-43 | 1.045229616 |
| Asthma | J45 | 1.024847372 |
| Atrial fibrillation and flutter | I48 | 1.071084957 |
| Cancer | C00-C76, C80-C97 | 1.614526599 |
| Cerebrovascular diseases | G45-G46, I60-I69 | 4.366433972 |
| Cerebrovascular stroke | I60-I69 | 4.339769592 |
| Congestive heart failure | I099, I110, I130, I132, I255, I420, I425-I429, I43, I50, P290 | 1.109509239 |
| Connective tissue disorder | M05-M06, M315, M32-M34, M351, M353, M36 | 1.007533029 |
| Dementia | F00-F04, F051, G30, G311 | 1.013943114 |
| Depression | F31-34 | 1.029095228 |
| Diabetes | E10-14, M14, N08 | 1.164134428 |
| Diabetic complications | E102-E105, E107, E112-E115, E117, E122-E125, E127, E132-E135, E137, E142-E145, E147 | 1.139024505 |
| Hypertension | I10-15, I67 | 1.098985732 |
| Ischemic heart diseases | I20-25 | 1.167677212 |
| Liver disease | B18, K700-K717, K73-K74, K76, Z944 | 1.005445392 |
| Lung cancer | C34 | 1.600050178 |
| Metastatic cancer | C77-C80 | 1.093350145 |
| Osteoporosis | M80-82 | 1.052310476 |
| Paraplegia | G041, G114, G801, G802, G81-G82, G830-G834, G839 | 1.006889935 |
| Peptic ulcer | K25-K28 | 1.008347355 |
| Peripheral vascular disease | I70-I71, I731, I738, I739, I771, I790, I792, K551, K558, K559, R02, Z958, Z959 | 1.025879615 |
| Other pulmonary disease | I278, I279, J40-J43, J46-47, J60-J67, J684, J701, J703 | 1.014078316 |
| Renal disease | I12, I13, N01, N03, N052-N057, N072-N074, N18, N19, N25, Z490-Z492, Z992 | 8.496715393 |
| Renal failure | N18-19 | 8.488893282 |
| Sleep apnoea | G4730 | 1.004138383 |

After finding the useful variables, we built and compared multiple models. Besides the version presented in the original manuscript, we replaced the CCI variable with individual comorbidities. In a third, fourth, and fifth model, we excluded the number of maintenance inhaled therapy days, the hospitalization number, and then both parameters from the original model, respectively. In order to find the statistically most relevant setup, we calculated the AIC for each model (also included as S4 Table in the Supporting information):

|  | **All-cause total cost** | **All-cause inpatient cost** | **COPD-specific total cost** | **COPD-specific inpatient cost** |
| --- | --- | --- | --- | --- |
| **Original model** | **1 370 963** | **561 377** | **1 119 811** | **151 013** |
| Model with individual comorbidities | 1 363 029 | 559 666 | 1 119 010 | 150 931 |
| Model without adjustment to maintenance inhaled therapy days | 1 373 959 | 561 658 | 1 172 853 | 151 010 |
| Model without adjustment to hospitalization number | 1 382 886 | 565 590 | 1 182 719 | 155 576 |
| Model without adjustment to maintenance inhaled therapy days and hospitalization number | 1 385 307 | 565 817 | 1 208 663 | 155 604 |

Model fit was best in the second model, where individual comorbidities were also considered. However, the variables related to the calendar year showed similar behaviour here as in the original model using CCI. Excluding the number of maintenance inhaled therapy days or the number of hospitalizations resulted in worse fitting models, except for COPD-specific inpatient costs, where the fit of the original and the model without adjusting to the therapy days is similar. We believe this supplementary analysis adequately addresses comments 9 and 10 raised by the reviewer, and do not support that the inclusion of maintenance inhaled therapy days and hospitalization number as co-variates. For comment 8, our additional analysis on a representative subsample shows that using individual comorbidities truly have a better fitted model.

Importantly, we add here the figures of the model with the individual comorbidities instead of the CCI. In line with the original results which showed that CCI is a major determinant of all-cause total and in-patient costs, we also found in the novel analysis that a wide range of comorbidities is linked with increasing costs. Among them, the effect of non-pulmonary cancer and metastatic cancer is the most prominent on the all-cause total and inpatient costs, while acute myocardial infarction shows also a marked positive associated with inpatients costs. However, regarding COPD-specific total costs the effect of comorbidities are much less, and there is a very modest positive association between costs and comorbidities (congestive heart failure, peptic ulcer, anxiety) which present symptoms potentially overlapping with those of COPD. Furthermore, no comorbidity increasing COPD-specific inpatients costs have been identified. Interestingly, concomitant diagnosis of cancer is linked with less COPD-related inpatient costs as COPD-related symptoms are possibly treated during hospital care primarily due to cancer treatment and not coded as hospitalization due to COPD.

However, in these models, the calendar years have similar effects as in our original model, therefore the message of our study is unaltered. As this analysis could not be performed in the whole data set, we kept the original figures in the main manuscript but expanded the section on “Statistical analysis” in the main text (lines 175-91), and in the Supporting information and included the results of this model in the Supporting information (S6-9 Figures) and mentioned them in the main text (lines 238-40, 253-5, 281-2, 338).

The association of clinical variables to all-cause total healthcare costs

The association of clinical variables to all-cause inpatient healthcare costs

The association of clinical variables to COPD-specific total healthcare costs

The association of clinical variables to COPD-specific inpatient healthcare costs
